# Conclusions of clinical trials assessing monoclonal antibodies and sponsored by pharmaceutical industry: a meta-research study

**DOI:** 10.1590/1806-9282.20241022

**Published:** 2024-12-02

**Authors:** Rachel Campos Ornelas, Débora de Souza Pazini, Rafael Leite Pacheco, Ana Luiza Cabrera Martimbianco, Rachel Riera

**Affiliations:** 1 Universidade Federal de Juiz de Fora – Governador Valadares (MG), Brazil.; 2 Hospital Sírio-Libanês – São Paulo (SP), Brazil.; 3 Centro Universitário São Camilo – São Paulo (SP), Brazil.; 4 Oxford-Brazil Evidence Based-Medicine Alliance – São Paulo (SP), Brazil.; 5 Universidade Metropolitana de Santos – Santos (SP), Brazil.; 6 Universidade Federal de São Paulo, Escola Paulista de Medicina – São Paulo (SP), Brazil.

**Keywords:** Antibodies, Monoclonal, Comparative effectiveness research, Drug industry, Clinical trial, Meta-analysis, Evidence-based medicine

## Abstract

**OBJECTIVE:**

The objective of this study was to describe and critically appraise the conclusions of randomized clinical trials assessing monoclonal antibodies sponsored by the pharmaceutical industry.

**METHODS:**

This is a meta-research study on conclusions’ characteristics of randomized clinical trials with monoclonal antibodies as interventions sponsored by the pharmaceutical industry.

**RESULTS:**

A total of 82 publications were considered. Notably, 79.3% (65/82) of the trials were fully funded by the pharmaceutical industry, and sponsors had data and publishing rights in 70.7% (58/82). Among the fully sponsored studies, 78.5% (51/65) presented conclusions with a drug-favorable direction of the effect, and 30.8% (20/65) made some recommendations for clinical practice, of which 95% (19/20) were favorable to the drug. In relation to the partially funded studies, drug-favorable direction of effect was present in 68.8% (11/16) of conclusions, and 18.8% (3/16) recommended the drug for the practice, in which 66.7% (2/3) favored the drug. Positive direction of effect was more present in trials in which the sponsor owned data and publication (81.0%; 47/58), compared to trials where the funder did not (63.3%; 14/22). Only 13.4% (11/82) of included studies recognize the uncertainty in the estimates in the conclusions, and 37.4% (31/82) had a statement regarding the need for future studies.

**CONCLUSIONS:**

Most of the included randomized clinical trials on monoclonal antibodies funded by the industry concluded a drug-favorable direction of effect. Recommendations for practice were common, while recognition of uncertainty and statements regarding the need for future studies were less frequently present in the conclusion.

## INTRODUCTION

Relying on bioethical principles, medical and other healthcare decision-makers should seek to do the best for their patients while pondering potential risks of conduct^1^. In this context, providing high-quality evidence research studies becomes essential, with randomized clinical trials (RCTs) occupying a leading position in reporting on treatments’ comparative efficacy and safety since such a methodological design seeks to remove confusion factors and assess causality^2^. Therefore, it is expected that the outcomes obtained from an RCT help to conclude whether there is superiority, non-inferiority, or equivalence in the conduct adopted, pointing out limitations and assisting in decision-making^2^.

Because of their complexity, RCTs are financially expensive^3^, often being financed, in whole or in part, by the pharmaceutical industry. If, on the one hand, such funding allows studies with higher methodological quality to be carried out, on the other hand, it increases the risk of sponsorship bias^4^. This bias corresponds to the influence of the study's design, conduct, and publication, so the sponsored product looks better than it is, thus promoting commercial interests^5^.

Recently, interest in biological agents, including monoclonal antibodies, has increased due to their multiple clinical applications in rheumatologic, neoplasia, and inflammatory diseases and their potential uses in other conditions^6^. RCT has been conducted to investigate monoclonal antibodies, including new therapies, and their effect on different medical conditions. Given the increasing number of clinical trials sponsored by the pharmaceutical industry, investigators might tend to publish conclusions with favorable outcomes for the sponsor when they summarize the findings of controlled clinical trials^7^.

## OBJECTIVE

The objectives of this study were to describe and critically appraise the conclusions of randomized clinical trials assessing monoclonal antibodies sponsored by the pharmaceutical industry.

## METHODS

### Design and setting

This meta-research study was carried out in the Discipline of Evidence-Based Medicine, Escola Paulista de Medicina (EPM), Universidade Federal de São Paulo (Unifesp), and Universidade Federal de Juiz de Fora (UFJF), Brazil. No validated guide for planning and conducting meta-research studies has been established in the literature. Therefore, this study will follow the recommendations proposed by Murad and Wang for reporting meta-epidemiological studies [Murad and Wang]^8^, in addition to the relevant items of the Preferred Reporting Items for Systematic Reviews and Meta-Analyses (PRISMA 2020) [Page et al]^9^, as has been proposed in the literature [Granholm et al]^10^.

### Criteria for including studies

We consider studies included in Medline between August 25, 2021, and March 03, 2022. We aimed to include the 80 most recent studies from the date of the search that fulfilled the eligibility criteria presented as follows. Therefore, we ordered the references retrieved from the search strategy and selected studies until we reached the sample size. As some studies were published on similar dates, we ended up including 82 studies. We did not conduct a sample size calculation, and this number was decided by convenience, but our intention was to include a recent and relevant sample of eligible studies.

#### Types of studies

We considered only randomized clinical trials fully or partially sponsored by the pharmaceutical industry, as declared in any relevant section of the publication.

#### Types of participants

Children and adults with a diagnosis of any condition, regardless of comorbidities. Healthy participants were not considered.

#### Types of intervention

All types of monoclonal antibodies were given as monotherapy or in combination. As comparators, we considered any active intervention (including biosimilar and radiation therapy), placebo, no intervention, or the same drug in different doses or administration routes.

### Outcomes of interest

The following outcomes were considered:

- What was the direction of the effect of the sponsored treatment? Totally favorable (toward a benefit), totally unfavorable (opposed to a benefit or toward harm), or neutral, considering the type of hypothesis.
- A recommendation for clinical practice was presented (yes, no).
- What was the direction of the recommendation for clinical practice? Totally favorable (toward a benefit), totally unfavorable (opposed to a benefit or toward harm), or neutral, considering the type of hypothesis.
- Is there a recognition of uncertainty? (yes, no). Examples of "yes" judgments are, but are not limited to, statements regarding sample size, study design limitations such as lack of blinding, high drop-out rates, and statements regarding confidence intervals.
- Is there a recognition that future studies are necessary? (yes, no).

The judgment was made based on the information obtained from at least one of the following article sections: the conclusion section in the main text, final considerations in the discussion section, and conclusions in the abstract.

### Search strategy

We searched randomized clinical trials indexed in MEDLINE (via PubMed on March 10, 2022) using the search strategy presented in Supplementary File 1.

### Studies election and data extraction

All references retrieved by the search strategy were collated in Microsoft Excel®. Two independent authors manually conducted the selection process to confirm the eligibility of studies. Two authors extracted the relevant information from the "Conclusion" section or the final considerations of the "Discussion" section. A third author was consulted in case of disagreements.

Aside from the outcomes of interest, the following general characteristics were extracted from the included studies:

### Author, publication date

- Clinical condition
- Number and names of pharma sponsors
- Extent of funding (full, partial, or unclear): we categorized the extension of the financing as declared by the authors from included studies
- Pharma with or without data and publishing rights (yes, no, unclear)
- Hypothesis/design of clinical trial: superiority, non-inferiority, or equivalence
- Type of comparator (placebo, drug, procedure, different doses, schemes or administration routes, biosimilar, no intervention)

### Data analysis and results presentation

Results were presented narratively or using tables and graphics when appropriate. Descriptive statistical analysis was performed using the STATA® software.

We analyzed the relationship of each outcome with two variables: the extent of funding (full/partial) or whether the pharma sponsor had publishing rights (yes/no) using chi-square tests. Our hypothesis was that studies fully sponsored or that pharma had publishing rights would be more likely to conclude a benefit and to make a recommendation for clinical price and less likely to recognize the uncertainty in the results. A pre-defined threshold of 0.05 was used to consider the association statistically significant.

## RESULTS

### Search results

The search strategy retrieved 10,452 references for screening. We evaluated 280 abstracts ordered by publication date until 102 studies met the eligibility criteria. Of these, 20 studies were excluded for reasons (Supplementary File 2), 15 did not meet the eligibility criteria, and 5 were unavailable. We extracted the results from the remaining 82 studies. The study selection process is presented as a flowchart in Supplementary File 3. We presented a list of the included studies in Supplementary File 4.

### Characteristics of included publications

The 82 studies included were published between 2021 and 2022. A summary of characteristics is presented in Table 1 (details of study characteristics are presented in Supplementary File 5). Detailed lists of clinical conditions and sponsors are shown in Supplementary Files 6 and 7.

**Table 1:** Summary of characteristics of the 82 included studies.

Item	Category	Frequency
Clinical condition (ICD-10)	Neoplasms	52.4% (43/82)
	Certain infectious and parasitic diseases	14.6% (12/82)
	Diseases of the musculoskeletal system and connective tissue	11.0% (9/82)
	Diseases of the skin and subcutaneous tissue	7.3% (6/82)
	Diseases of the respiratory system	4.9% (4/82)
	Endocrine, nutritional, and metabolic diseases	2.4% (2/82)
	Congenital malformations, deformations, and chromosomal abnormalities	1.2% (1/82)
	Diseases of the circulatory system	1.2% (1/82)
	Diseases of the digestive system	1.2% (1/82)
	Diseases of the genitourinary system	1.2% (1/82)
	Diseases of the nervous system	1.2% (1/82)
	Factors influencing health status and contact with health services	1.2% (1/82)
Pharma sponsor*	Roche	18.3% (15/82)
	Bristol Myers Squibb	13.4% (11/82)
	Lilly	8.5% (7/82)
	Merck	8.5% (7/82)
	Regeneron Pharmaceuticals	8.5% (7/82)
	Ono Pharmaceutical	6.1% (5/82)
	Sanofi	6.1% (5/82)
	Amgen	4.9% (4/82)
	Janssen	4.9% (4/82)
	Jiangsu Hengrui	4.9% (4/82)
	JSC BIOCAD	3.7% (3/82)
	Pfizer	3.7% (3/82)
Number of sponsors	5	1.2% (1/82)
	2	24.4% (20/82)
	1	74.4% (61/82)
Hypothesis	Superiority	90.2% (74/82)
	Equivalence	8.5% (7/82)
	Unclear	1.2% (1/82)
Type of comparator	Schedule A vs. schedule B	2.4% (2/82)
	Drug+treatment x vs. treatment x	14.6% (12/82)
	Drug vs. placebo	52.4% (43/82)
	Drug vs. similar	8.5% (7/82)
	Drug x vs. drug y	22.0% (18/82)
Extent of funding	Full	79.3% (65/82)
	Partial	19.5% (16/82)
	Unclear	1.2% (1/82)
Pharma sponsor had data and publishing rights	Yes	70.7% (58/82)
	No	26.8% (22/82)
	Unclear	2.4% (2/82)

* Industries that sponsored at least three studies. A full list is presented in the Supplementary File 6. ICD-10: International Classification of Diseases-10.

Thirty-seven clinical conditions were studied in the analyzed studies. COVID-19 was the most studied clinical condition (n=10, 12.2%), and cancer was the most studied group (n=43, 52.4%). 36 different industries funded the studies. Of these, 12 funded at least three studies, and the others funded up to 2. Roche sponsored the largest studies (n=15, 18.3%). 90.2% (n=74) presented a superiority hypothesis, and 52.4% (n=43) had a placebo as a comparator. Most studies had full funding (n=65; 79.3%), and their funder had the data and decided the publication (n=58; 70.7%).

### Outcomes

The outcome frequencies are presented in Table 2. A total of 63 (76.8%, 63/82) studies showed a totally favorable effect of the treatment in conclusion, and 28.0% (23/82) made a recommendation for clinical practice. Of these, 21 (91.3%, 21/23) presented a totally favorable both effect and recommendation of the treatment, and the remaining (8.7%, 2/23 was totally unfavorable). Most studies did not recognize uncertainty in the conclusion (86.6%, 71/82), nor the necessity nor not of future studies (51/82; 62.6%).

**Table 2:** Outcomes of interest.

Item	Category	Frequency
Direction of the effect	Totally favorable	76.8% (63/82)
	Totally unfavorable	22.0% (18/82)
	Neutral	1.2% (1/82)
Recommendation for clinical practice	Yes	28.0% (23/82)
	No	72.0% (59/82)
Direction of recommendation for clinical practice	Totally favorable	91.3% (21/23)*
	Totally unfavorable	8.7% (2/23)*
Recognition of uncertainty	Yes	13.4% (11/82)
	No	86.6% (71/82)
Recognition of necessity or not of future studies	Yes	37.8% (31/82)
	No	62.2% (51/82)

* Denominators are the number of studies (n=23) that presented a recommendation for clinical practice in the conclusion.

Despite only the association between recognition of the need for future studies and whether the industry had data and publishing rights showing a statistically significant association, the summarized results from Table 3 provide valuable insights.

**Table 3:** Relationship between the outcome of interest and the extent of funding or ownership of data and publication rights.

Outcome	Extent of funding		RR (95%CI; p)	Pharma sponsor had data and publishing rights		RR (95%CI; p)
	Full (n=65)	Partial (n=6)		Yes (n=58)	No (n=22)	
Favorable direction of the effect	78.5% (51/65)	68.8% (11/16)	1.14 (0.80–1.63); p=0.4115	81.0% (47/58)	63.6% (14/22)	1.27 (0.91–1.79); p=0.1025
Presence of recommendation for clinical practice	30.8% (20/65)	18.8% (3/16)	1.64 (0.56–4.85); p=0.3395	31.0% (18/58)	22.7% (5/22)	1.37 (0.58–3.23); p=0.4635
Favorable direction of recommendation for clinical practice	95.0% (19/20)	66.7% (2/3)	1.42 (0.64–3.20); p=0.1044	100.0% (18/18)*	60.0% (3/5)*	1.67 (0.82–3.41); p=0.0050
Recognition of uncertainty	10.8% (7/65)	25.0% (4/16)	0.43 (0.14–1.29); p=0.1366	12.1% (7/58)	18.2% (4/22)	0.66 (0.21–2.05); p=0.4784
Recognition of necessity or not of future studies	33.8% (22/65)	56.3% (9/16)	0.60 (0.35–1.04); p=0.0986	25.9% (15/58)	68.2% (15/22)	0.38 (0.23–0.64); p=0.0005

* Denominators are the number of studies that presented a recommendation for clinical practice in the conclusion. RR: risk ratio; CI: confidence interval.

## DISCUSSION

This meta-research study assessed 82 randomized clinical trials related to monoclonal antibodies and sponsored by the pharmaceutical industry. Most trials evaluated the effects of the intervention on neoplasm participants (52.4%), and COVID-19 was a predominantly studied clinical condition (12.2%). Placebo was the most frequent comparator group (52.4%), and almost all studies had superiority hypotheses (90.2%).

Generally, 79.3% of the studies were fully funded, and 70.7% of the funders maintained data and publishing rights. Among the fully sponsored trials, 78.5% presented a conclusion with a drug-favorable direction of the effect, and of the 30.8% that proposed some recommendation for clinical practice, 95% favored the drug. Regarding the studies with partial funding, 68.8% concluded with a drug-favorable direction of effect, and among the 18.8% who recommended the practice, 66.7% favored the drug. Furthermore, the direction of effect was positive in 81% of studies where the funder owned data and publication rights, compared to 63.3% in studies where the funder did not. In summary, there was a higher frequency of direction of effect and favorable treatment recommendation in fully funded studies or in which the funder had the data and decided to publish, compared to partially funded studies or in which the funder did not have data nor chose publication. Concerning recognizing uncertainties inherent to the study conduct, it was reported in 1 in 10 fully funded studies and one in four partially financed studies. In addition, the association between pharma sponsor-owned data and publishing rights and recognition of the need for further studies was significant.

In the sample included, studies that had full funding or whether the industry had data and publishing rights were more likely to provide a clear recommendation for practice and less likely to state the need for future studies.

This raises the question of whether the conclusion of a randomized controlled trial is the right place to perform a clinical practice recommendation or if this type of statement should be performed in a more contextualized scenario, such as in a clinical practice guideline. This is more worrisome because the recognition of uncertainty in the conclusion was very low in the entire sample. If clinical trials would make a clinical recommendation in their conclusions, a statement regarding uncertainty in the estimates must be presented.

A previous study^11^ investigating whether industry-funded comparative trials have their design and results affected showed that those studies seem to be larger, had their protocols more commonly registered, had higher citation impact, and produced beneficial findings to the sponsoring companies more often than trials supported by not-for-profit organizations. Another study^4^ compared clinical trials on lung cancer treatment sponsored by the pharmaceutical industry versus other sources, and it was observed that those funded could be published more easily in open-access journals, increasing the visibility of these studies and consequently widely disseminating their findings.

A similar study^12^ analyzed the association between funding and the conclusions of randomized clinical trials regarding the benefit of treatment. It also reported that conclusions are frequently positive for the experimental drug and more likely to recommend it if for-profit organizations funded the study. This fact may be explained by the lack of recognition of uncertainties and potential biases related to reporting and publication, for example, choosing only positive results or early interrupting trials with unfavorable findings.

Our study has limitations. First, the data collected was based on the reports found in articles about funding and data sharing, limited by interpretation and the formatting requirements of the journals in which the studies were submitted. Additionally, the period analyzed was short, making it impossible to evaluate changes over time in the impact of industry funding. Another limitation was the absence of an analysis of dissemination bias. Statistically positive results are more likely to be published, and there may have been a selection of the proposed initial outcomes^13,14^. Therefore, we cannot infer that industry funding interfered with the presentation of the results of the evaluated studies. As strengths, this is the first analysis of conclusions from randomized trials sponsored by the pharmaceutical industry that assessed therapies with monoclonal antibodies, including the comparison between partial and complete funding.

The results of the present study reinforce the requirement to improve the reporting of the sources of funding of a clinical trial, whether total or partial, and the implementation of mechanisms that increase transparency in the data acquisition and sharing so that it is possible to analyze the study more objectively and identify potential biases so that clinical decisions are made more accurately. Additionally, it is necessary to improve the reporting of conclusions consistent with the results found in the study, mainly derived from clinically relevant outcomes, and recognize the probable uncertainties related to the planning and conduct of the clinical trial and potential conflicts of interest. Future research needs to systematize and report more clearly about the ownership of data used in randomized controlled trials and how funding was used.

## CONCLUSION

Most of the included randomized clinical trials on monoclonal antibodies funded by the industry concluded a drug-favorable direction of effect. Recommendations for practice were common, while recognition of uncertainty and the need for future studies were less frequently present in the conclusion. In general, the characteristics of the conclusions were similar between total and partially funded studies and between studies where the industry declared or did not have the right to publish or share the data.

## Data Availability

All data are provided in the supplementary files fully available at https://github.com/rlpacheco/coi_monoclonal_antibodies-.git.
